# Supplementary material for: A Polyphasic Approach including Whole Genome Sequencing Reveals Paecilomyces paravariotii sp. nov. as a Cryptic Sister Species to P. variotii
Source: J Fungi (Basel). 2023 Feb 22;9(3):285. doi: 10.3390/jof9030285 (PMC10055108; doi:10.3390/jof9030285)
Supplement: Supplementary file 1 [file jof-09-00285-s001.zip › jof-2140383-supplementary.pdf]

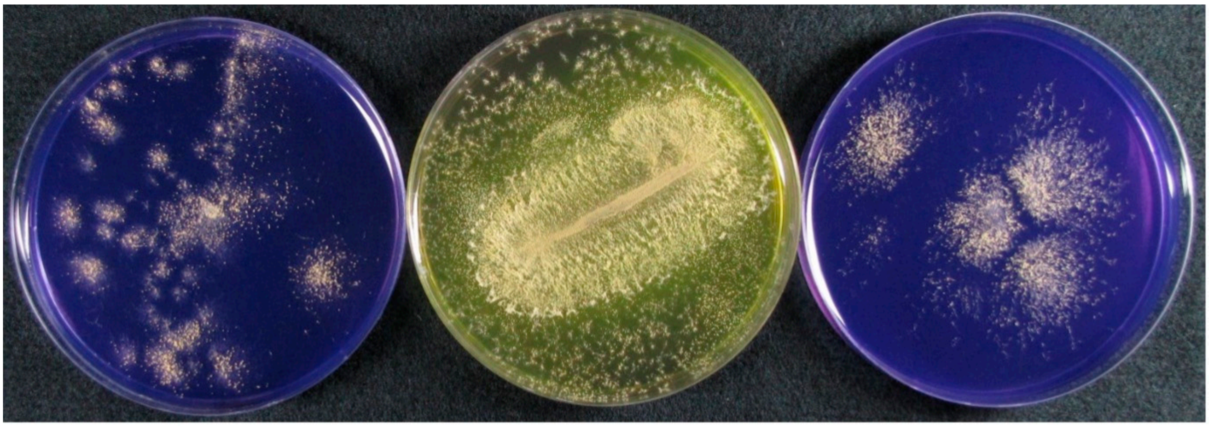

Figure S1. Acid production, indicated by yellow colour of pH indicator, of (left to right) *P. variotii* CBS 101075, *P. lecythidis* FRR 4481 and *P. paravariotii* FRR 5287.

Table S1. Genome assembly statistics.

Strain	Contigs	Total size (Mbp)	N50 (bp)
P. fulvus FRR 3794	76	31.5	835778
P. dactylethromorphus FRR 0699	64	34.9	1960473
P. dactylethromorphus FRR 5262	43	34.6	2439234
P. lecythidis FRR 3797	43	31.9	3019978
P. lecythidis FRR 4481	31	32.0	3687581
P. formosus FRR 3793	42	29.6	1721955
P. maximus FRR 4742	131	33.4	1401211
P. maximus FRR 2337	147	30.9	1212462
P. maximus FRR 4140	151	31.1	1305261
